# Orexin A affects HepG2 human hepatocellular carcinoma cells glucose metabolism via HIF-1α-dependent and -independent mechanism

**DOI:** 10.1371/journal.pone.0184213

**Published:** 2017-09-08

**Authors:** Xing Wan, Yuanyuan Liu, Yuyan Zhao, Xiaoqi Sun, Dongxiao Fan, Lei Guo

**Affiliations:** 1 Department of Medical Oncology, First Affiliated Hospital, China Medical University, Shenyang, Liaoning, P.R. China; 2 Department of Endocrinology, First Affiliated Hospital, China Medical University, Shenyang, Liaoning, P.R. China; 3 Department of Orthopedic Surgery, First Affiliated Hospital, China Medical University, Shenyang, Liaoning, P.R. China; University of Nebraska Medical Center, UNITED STATES

## Abstract

Orexins are hypothalamic neuropeptides that regulate feeding, reward, wakefulness and energy homeostasis. The present study sought to characterize the involvement of orexin A in glucose metabolism in HepG2 human hepatocellular carcinoma cells, and investigated the role of hypoxia-inducible factor-1α (HIF-1α) in the response. HepG2 cells were exposed to different concentrations of orexin A (10^−9^ to 10^−7^ M) *in vitro*, without or with the orexin receptor 1 (OX1R) inhibitor (SB334867), HIF-1α inhibitor (YC-1) or a combination of both inhibitors. Subsequently, OX1R, HIF-1α expression and localization, glucose uptake, glucose transporter 1 (GLUT1) expression and ATP content were measured. We further investigated the intracellular fate of glucose by measuring the gene expression of pyruvate dehydrogenase kinase 1 (PDK1), lactate dehydrogenase (LDHA) and pyruvate dehydrogenase B (PDHB), as well as metabolite levels including lactate generation and mitochondrial pyruvate dehydrogenase (PDH) activity. The activity of phosphoinositide 3-kinase (PI3K)/Akt/mammalian target of rapamycin (mTOR) pathway was also assessed. Our results showed that the expression of OX1R was predominantly located in the nucleus in HepG2 cells. Orexin A oxygen-independently promoted the mRNA and protein expression of HIF-1α as well as its nuclear accumulation in HepG2 cells and the elevated HIF-1α protein was associated, at least partly, with the activation of the PI3K/Akt/mTOR pathway. Orexin A stimulated GLUT1 expression, glucose uptake as well as ATP generation in HepG2 cells via OX1R acting through the HIF-1α pathway. Moreover, orexin A inhibited LDHA, PDK1 expression and lactate production, stimulated PDHB expression and PDH enzyme activity independent of HIF-1α. Our results indicated that orexin signaling facilitated the glucose flux into mitochondrial oxidative metabolism rather than glycolysis in HepG2 cells. These findings provide new insight into the regulation of glucose metabolism by orexin A in hepatocellular carcinoma cells.

## Introduction

Orexin A and orexin B are a pair of hypothalamic neuropeptides that play critical roles in the regulation of feeding, wakefulness, reward system, energy homeostasis as well as other physiological process [1–4]. The actions of orexin peptides are mediated via interaction with two G protein-coupled receptors (GPCRs), orexin receptor types 1 and 2 (OX1R and OX2R, respectively). OX1R has a higher selectivity for orexin A, while OX2R binds both orexin peptides with similar affinity [5]. In addition to hypothalamus, orexins and their receptors are also widely expressed in peripheral tissues, such as adrenal gland, adipose tissue, digestive tract, and gonads [6, 7]. Orexin singling deficiency caused narcolepsy in human and animal models, which was accompanied by metabolic abnormalities, such as elevated incidence of obesity and type 2 diabetes [8, 9]. Recent study demonstrated that transgenic orexin overexpression efficiently protected mice from insulin resistance induced by high fat diet [10], whereas the deletion of orexin caused disarrangement of hepatic insulin signaling and abnormal gluconeogenic activity during ageing [11, 12], suggesting an important role of orexin in glucose metabolism.

Despite the central significance of orexin system in regulating energy and glucose metabolism, little is known about the metabolic effects of orexin on cancer cells. It has been recognized that the metabolic processes of tumor cells are different from that of their normal counterparts. Malignant tumor cells exhibit significantly increased glucose uptake to meet their high energy demands [13]. Moreover, unlike normal differentiated cells, which metabolize glucose preferentially through mitochondrial oxidative phosphorylation for ATP generation, most cancer cells reprogram the metabolic flux towards glycolysis even in the presence of enough oxygen, leading to a state termed “Warburg effect” or “aerobic glycolysis” [14, 15]. This metabolic switch results in anabolic and bioenergetics changes that provide a growth advantage for cancer cells [16, 17], and has been regarded as a critical hallmark of cancer.

Hypoxia-inducible factor-1 (HIF-1), a major transcription factor responsible for cellular adaptation to hypoxia [18], has been implicated in the central regulation of cancer-specific glucose metabolism. HIF-1 is a heterodimer consisting of HIF-1α and HIF-1β. HIF-1β is a constitutively expressed subunit, whereas HIF-1α is an oxygen-sensitive subunit that determines the activity of HIF-1 [19]. Under normoxia, HIF-1α is hydroxylated by proline hydroxylase domain (PHD) proteins, enabling the von Hippel-Lindau (VHL) to recognize and target HIF-1α for ubiquitination and proteasomal degradation [20]. Hypoxia inhibits the activity of PHD and prevents HIF-1α from degradation. HIF-1α thus accumulates and imports into the nucleus, where it forms the heterodimer with HIF-1β, triggering the transcription of diverse genes involved in cell survival, angiogenesis, invasion and cell metabolism [21]. Specifically, the HIF-1 plays a role in regulating intracellular glucose metabolism. By promoting the expression of glucose transporter 1 (GLUT1) and glycolytic enzymes, HIF-1 leads to increased glucose uptake and glycolysis [22]. Moreover, HIF-1 helps to shunt the pyruvate flux into glycolysis rather than tricarboxylic acid (TCA) cycle and oxidative phosphorylation. This is due, at least partly, to the fact that HIF-1 stimulates the expression of lactate dehydrogenase A (LDHA) [21], the enzyme that catalyzes pyruvate to lactate, as well as pyruvate dehydrogenase kinas (PDK) [23, 24], which phosphorylates and inactivates pyruvate dehydrogenase (PDH), the enzyme that processes pyruvate to acetyl-CoA. Certain studies have reported that orexin A can induce the expression of HIF-1α under normoxic conditions [25–27]. However, little is known about the ability of this peptide to regulate HIF-1α in hepatic carcinoma cells.

Recently, the expression of orexin receptors have been detected in a set of tumor cell lines [28–35], signifying a potential role of this peptide in tumor biology. However, the potential role of orexin A and its receptors in hepatic carcinoma cells is unclear. In this study, we sought to examine the expression of orexin receptors in HepG2 human hepatocellular carcinoma cells, and further investigated whether the orexin A/OX1R-stimulated HIF-1α signaling mediated its effects on glucose metabolism in HepG2 cells.

## Materials and methods

### Reagents

Orexin A was obtained from Sigma-Aldrich (St. Louis, MO, USA). Dulbecco's modified Eagle's medium (DMEM) and fetal bovine serum were purchased from Gibco (Grand Island, NY, USA). Cobalt chloride (CoCl_2_), Akt inhibitor (LY294002), mTOR inhibitor (temsirolimus) and HIF-1α inhibitor (YC-1) were purchased from Sigma-Aldrich (St. Louis, MO, USA). OX1R inhibitor (SB334867) was purchased from Tocris Bioscience (Minneapolis, MA, USA). Total/phosphor-Akt polyclonal antibody, total-mTOR polyclonal antibody, phosphor-mTOR monoclonal antibody, GLUT1 monoclonal antibody, OX1R polyclonal antibody, OX2R polyclonal antibody and β-actin polyclonal antibody were all obtained from Abcam (Cambridge, MA, USA). HIF-1α polyclonal antibody was obtained from Novus Biologicals (Littleton, CO, USA).

### Cell culture

Human hepatocellular carcinoma cell line HepG2 was obtained from the Type Culture Collection of the Chinese Academy of Sciences (Shanghai, China) and maintained in DMEM medium supplemented with 10% fetal bovine serum, 50 μg/ml penicillin and 100 μg/ml streptomycin (Xianfeng, Shanghai, China). The cells were grown in a humidified atmosphere containing 5% CO_2_ at 37°C. Before the experiment, the cells were grown in petri dishes in a serum-free medium for 24 h. The following day, the cells were treated with different concentrations of orexin A (0, 10^−9^, 10^−8^ and 10^−7^ M). Another group of HepG2 cells was incubated without or with 10^−6^ M SB334867 (OX1R inhibitor), 10^−5^ M YC-1 (HIF-1α inhibitor), or a combination of both inhibitors prior to stimulation with 10^−7^ M orexin A.

### Measurement of glucose uptake

HepG2 cells were cultured overnight in serum-free DMEM. Cells were rinsed twice with phosphate-buffered saline (PBS) and incubated in glucose-free Krebs–Ringer–HEPES (KRH) buffer (25 mM Hepes, pH 7.4, 1.3 mM CaCl_2_, 120 mM NaCl, 1.2 mM MgSO_4_, 1.3 mM KH_2_PO_4_ and5 mM KCl) with the indicated concentrations of orexin A with or without the OX1R inhibitor, HIF-1α inhibitor, or a combination of both inhibitors. After 30min, 0.5 μCi of2-Deoxy-D [^3^H] glucose (2-DG) (PerkinElmer Life Sciences) was added for 15 min at 37°C. Subsequently, ice-cold PBS was used to terminate the reaction. Cells were solubilized for 10 min in 0.1% sodium dodecyl sulfate (SDS). Aliquots of cell lysates were used for liquid scintillation. Protein concentration was measured using a BCA protein assay reagent kit (Beyotime Institute of Biotechnology, Shanghai, China). Data were normalized to protein concentration and showed as percentage of basal uptake.

### Measurement of cellular ATP

HepG2 cells were exposed to orexin A at concentrations of 0, 10^−9^, 10^−8^ and 10^−7^ M for 2 h. Another group of cells was incubated without or with the OX1R inhibitor, SB334867 (10^−6^ M), HIF-1α inhibitor, YC-1 (10^−5^ M), or a combination of both inhibitors for 30 min prior to stimulation with 10^−7^ M orexin A for 2 h. ATP content was measured using a ATP Assay kit (Beyotime) according to the manufacturer’s instructions.

### Immunofluorescence

HepG2 cells were fixed with 4% paraformaldehyde (Solarbio, Beijing, China) for 30 min, washed with PBS, and permeabilized in 0.1% Triton X-100 (Solarbio) for 30 min. Subsequently, the cells were blocked with 5% bovine serum albumin (BSA, Solarbio) for 30 min at room temperature and then incubated with HIF-1α antibody (1:100, Novus Biologicals) overnight at 4°C. The next day, cells were washed with PBS, incubated with fluorescein isothiocyanate (FITC)-labeled goat anti-rabbit IgG (H+L) secondary antibody (1:100, ZSGB-BIO, Beijing, China) for 30 min at room temperature. Cells were rinsed with PBS, counterstained with 4’, 6-diamidino-2-phenylindole (DAPI) (Solarbio) for 5 min, and visualized with a fluorescence microscope (Olympus).

### Measurement of lactate

HepG2 cells were incubated with or without 10^−7^ M orexin A for 0, 2, 4 and 8 h in the presence or absence of OX1R inhibitor, SB334867 (10^−6^ M) and HIF-1α inhibitor, YC-1 (10^−5^ M). Medium from cultured cells was collected and lactate content was measured using a Lactic Acid assay kit (Nanjing Jiancheng Bioengineering Institute, Nan Jing, China) according to the manufacturer’s instructions.

### Pyruvate dehydrogenase activity

HepG2 cells were incubated with or without 10^−7^ M orexin A for 0, 15, 30, 60 and 120 min in the presence or absence of OX1R inhibitor, SB334867 (10^−6^ M) and HIF-1α inhibitor, YC-1 (10^−5^ M). Mitochondria was isolated as previously described [36]. PDH activity in mitochondrial lysate was determined by a Pyruvate dehydrogenase(PDH) Enzyme Activity Microplate Assay Kit (Abcam, Cat. no. ab109902) according to the manufacturer’s instructions.

### Real-time PCR

Total RNA was extracted from HepG2 cells using TRIzol reagent (Life Technologies Co., Carlsbad, CA, USA). After spectrophotometric quantification, 1 μg total RNA was converted into cDNA using the PrimeScript^™^ RT reagent kit with gDNA Eraser (Takara Bio, Otsu, Japan) according to the manufacturer's instructions. Aliquots of cDNA corresponding to equal amounts of RNA were used for the quantification of mRNA by real-time PCR using the Light Cycler 96 real-time quantitative PCR detection system (Roche, Indianapolis, IN, USA). Specific primers were used as follows:HIF-1α forward, 5’-CAA GAA CCT ACT GCT AAT GCC ACC-3’ and reverse, 5’-GTA TGT GGG TAG GAG ATG GAG ATG-3’; PDK1 forward, 5’-CTC AAG TAA TCC TTC CAC CTC AGC-3’ and reverse, 5’-GGA ACA TAC TGA GAC CTC ATC TCC-3’; LDHA forward, 5’-ATC TTG ACC TAC GTG GCT TGG A-3’ and reverse, 5’-CCA TAC AGG CAC ACT GGA ATC TC -3’; PDHB forward, 5’-GTG ATA AAT ATG CGT ACC ATT AGA CC-3’ and reverse, 5’-CAG CAC CAG TGA CAC GAA CAG C-3’. As an internal control for reverse transcription (RT) and reaction efficiency, amplification of glyceraldehyde-3-phosphate dehydrogenase (GAPDH) mRNA was carried out in parallel for each sample. The specific primers used were: GAPDH forward, 5’-TGA AGG TCG GAG TCA ACG G-3’ and reverse, 5’-CCT GGA AGA TGG TGA TGG G-3’. The reaction system was 25 μl, including 2 μl cDNA template, 2 μl forward and reverse primers, 8.5 μl RNase-free ddH_2_O, and 12.5 μl SYBR^®^ Premix Ex Taq^™^ II (Takara). The PCR reactions were carried out using the following conditions: 95°C for 30 sec, then 40 cycles of 95°C for 5 sec, 60°C for 30 sec, and 95°C for 15 sec. All primers specific to HIF-1α, PDK1, LDHA, PDHB and GAPDH were designed using Primer Premier 5.0 software (Premier Biosoft International, Palo Alto, CA, USA).

### Protein preparations and western blot analysis

Total protein was extracted from HepG2 cells using radioimmunoprecipitation assay cell lysis reagent containing proteinase and phosphatase inhibitors (Solarbio). The cells remained in the medium on ice for 30 min with re-dispersion every 5 min. Cell lysates were centrifuged at 12,000 x g for 10 min at 4°C, and the protein concentrations of the supernatants were determined using the BCA protein assay reagent kit (Beyotime). The supernatants containing total protein were mixed with a corresponding volume of 5X SDS loading buffer and were subsequently denatured by boiling for 10 min. Samples were separated by sodium dodecyl sulfate polyacrylamide gel electrophoresis (SDS-PAGE) using 5% stacking and 12% separating gels. Subsequently, the samples were transferred onto polyvinylidene difluoride (PVDF) membranes (0.2 μm, Immobilon-P; Millipore, Billerica, MA, USA) at 60 V for 2.5 h. After blocking with skimmed dry milk for 2 h at room temperature, the membranes were washed three times with Tris-buffered saline with Tween 20 (TBST) for 30 min. Then they were incubated overnight at 4°C with the appropriate primary antibody. The primary antibodies and dilutions used were as follows: rabbit anti-human OX1R (cat. no. ab68718, 1:1000), rabbit anti-human OX2R (cat. no. ab129432, 1:500), rabbit anti-human Akt (cat. no. ab8805, 1: 1,000), rabbit anti-human phospho-Akt (cat. no. ab8932, 1: 1,000), rabbit anti-human mTOR (cat. no. ab2732, 1: 2,000), rabbit anti-human phospho-mTOR (cat. no. ab109268, 1: 1,000), rabbit anti-human HIF-1α (cat. no. NB100-479, 1:1000), rabbit anti-human GLUT1 (cat. no. ab115730, 1: 1,000), rabbit anti-human β-actin (cat. no. ab8227, 1: 1,000). The membranes were washed and incubated at room temperature for 1.5 h with the secondary goat anti-rabbit lgG (H+L) antibody (Beyotime, cat. no. A0208, 1: 2,000) conjugated with horseradish peroxidase, then washed three times with TBST for 30 min. The membranes were subjected to enhanced chemiluminescence (ECL) using an ECL detection kit (Beyotime, cat. no. P0018) and quantified using Quantity One software (Bio-Rad Laboratories Inc., Hercules, CA, USA).

### Statistical analysis

The results were expressed as means ± standard error of the mean and differences between the means were analyzed by one-way or two-way analysis of variance (ANOVA) where appropriate. *P<0*.*05* was considered to be statistically significant. Statistical analysis was performed using the SPSS 15.0 software package (SPSS Inc., Chicago, IL, USA).

## Results

### OX1R expression and localization in HepG2 cells

**Fig 1:**
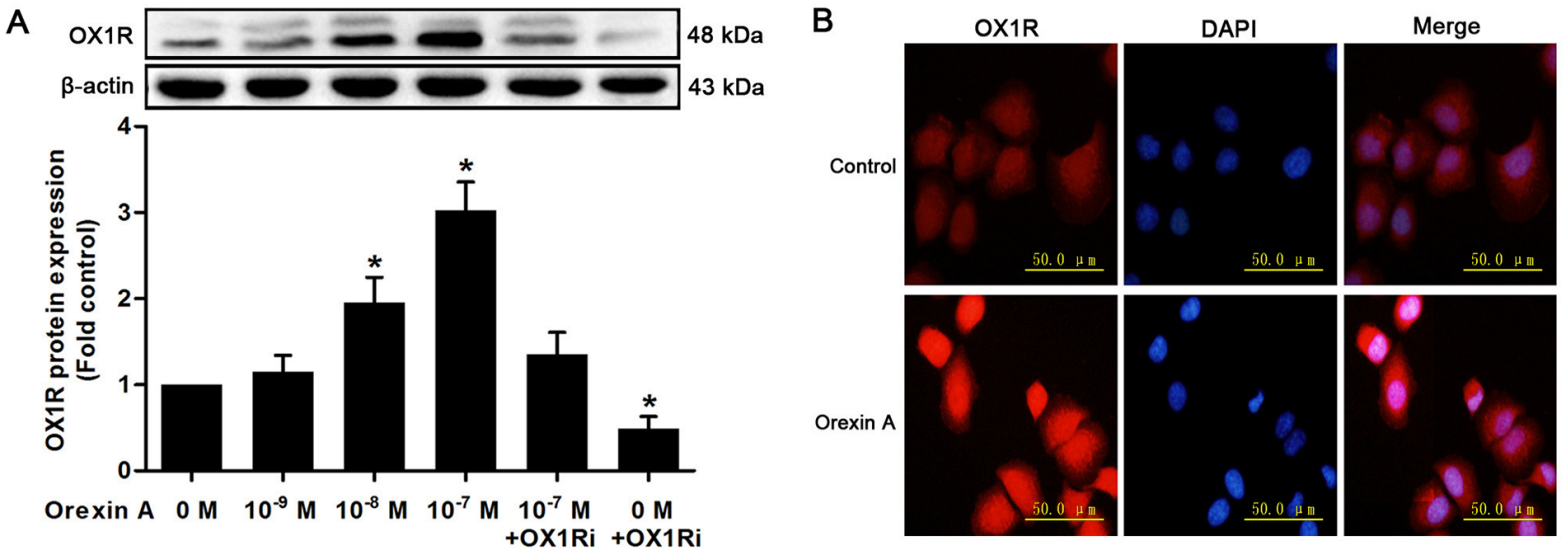
Orexin receptor 1 (OX1R) expression and localization in HepG2 human hepatocellular carcinoma cells. (A) HepG2 cells were incubated with orexin A at concentrations of 0, 10^−9^, 10^−8^ and 10^−7^ M for 24 h with or without the OX1R inhibitor SB334867 (10^−6^ M). OX1R protein expression was determined by western blot analysis. Data are presented as mean ± standard error of the mean based on analysis in triplicate. *P< 0.05 compared to control. (B) HepG2 cells were cultured with or without 10^−7^ M orexin A for 24 h. The cellular localization of OX1R was measured by immunofluorescent staining using the anti-OX1R antibody. Nucleus was immunolabeled with 4′,6-diamidino-2-phenylindole dihydrochloride (DAPI). Staining was analyzed by fluorescence microscope.

We first performed western blot to assess the protein expression of orexin receptors in HepG2 cells. The expression of OX1R was detected in HepG2 cells. Moreover, orexin A stimulation for 24 h resulted in a dose-dependent increase in OX1R expression, with the highest response, 3.02-fold compared to the control, observed at 10^−7^ M (Fig 1A). These effects were blocked with 10^−6^ M SB334867, a specific OX1R inhibitor (Fig 1A). We did not detect the expression of OX2R in HepG2 cells.

Next, we investigated the cellular distribution of OX1R protein in HepG2 cells by immunofluorescent staining. The OX1R-immunopositivity was predominantly located in the nucleus. OX1R was also present in cytoplasm and plasma membrane. Incubation of the cells with orexin A for 24 h resulted in a clear increase in overall OX1R labeling (Fig 1B). These results indicated that OX1R was expressed in HepG2 hepatoma cells and exhibited to be highly sensitive to exogenous orexin A stimulation.

### Orexin A-mediated induction of HIF-1α

**Fig 2:**
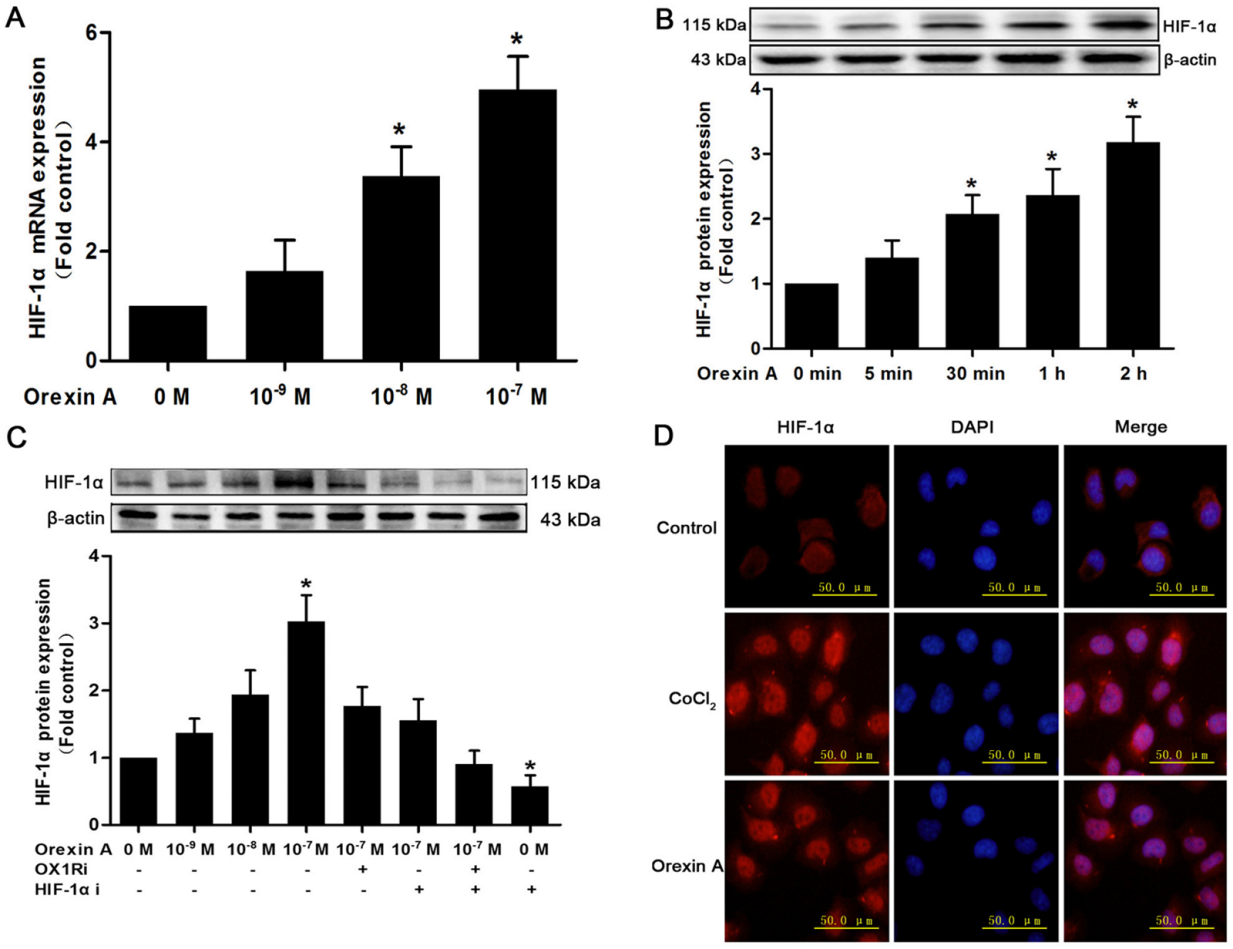
Effects of orexin A on HIF-1α expression and intracellular localization in HepG2 cells. (A) HepG2 cells were treated with the indicated concentrations of orexin A for 2 h. HIF-1α mRNA expression was measured by real-time PCR. (B) HepG2 cells were treated with 10^−7^ M orexin A for the indicated times. HIF-1α protein expression was measured by western blot. (C) HepG2 cells were treated with the indicated concentrations of orexin A for 2 h with or without the OX1R inhibitor, SB334867 (10^−6^ M), HIF-1α inhibitor, YC-1 (10^−5^ M), or a combination of both inhibitors. HIF-1α protein expression was measured by western blot. Data are presented as mean ± standard error of the mean based on analysis in triplicate. *P< 0.05 compared to control. (D) HepG2 cells were treated without or with 10^−7^ M orexin A under normoxia for 2 h, or 150 uM CoCl_2_ (stimulating hypoxia) for 8 h. The cellular localization of HIF-1α was measured by immunofluorescent staining using the anti-HIF-1α antibody. Nucleus was immunolabeled with 4′, 6-diamidino-2-phenylindole dihydrochloride (DAPI). Staining was analyzed by fluorescence microscope.

Given the role of HIF-1α in glucose metabolism and cancer progression, we investigated several levels of HIF-1α regulation in HepG2 cells stimulated with orexin A. Real-time PCR assays demonstrated that the mRNA level of HIF-1α was dose-dependently increased in response to orexin A treatment. The effects of 10^−8^ M and 10^−7^ M orexin A were statistically significant, increasing HIF-1α mRNA expression by 3.38- and 4.95-fold compared with the control, respectively (Fig 2A). As regards protein expression, a basal level of HIF-1α was seen in normoxic HepG2 cells. Incubation of the cells with orexin A under normoxia resulted in time- and dose-dependent induction of HIF-1α (Fig 2B and 2C). Treatment with 10^−7^ M orexin A for 2 h exerted the most potent effect, increasing HIF-1α protein expression by 3.02-fold over basal. Moreover, pretreatment with the OX1R inhibitor SB334867 (10^−6^ M) and HIF-1α inhibitor YC-1 (10^−5^ M) for 30 min effectively reduced the elevated HIF-1α protein stimulated by orexin A (Fig 2C).

We next performed immunofluorescence analysis to investigate the intracellular localization of HIF-1α in HepG2 cells upon orexin A stimulation. As shown in Fig 2D, HIF-1α could be weakly detected in HepG2 cells in normoxia, while a distinct increase of nuclear staining of HIF-1α was observed in cells treated with CoCl_2_ (stimulating hypoxia). Exposure of the cells with orexin A under normoxia also induced HIF-1α protein to accumulate in the nucleus. Collectively, these results indicated that orexin A promoted the mRNA and protein expression of HIF-1α as well as its nuclear accumulation in HepG2 cells under normoxia.

### PI3K/Akt/mTOR signaling pathway is involved in orexin A-mediated induction of HIF-1α

**Fig 3:**
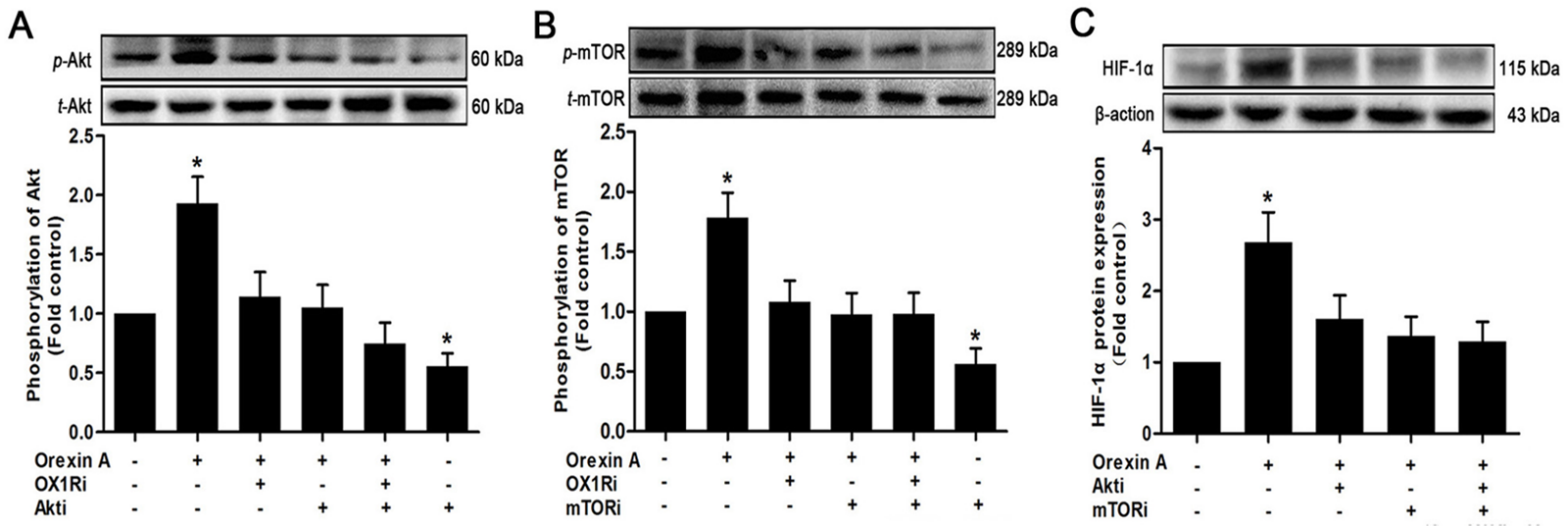
Effects of PI3K/Akt/mTOR signaling on orexin A-induced HIF-1α protein accumulation. HepG2 cells were treated with or without 10^−7^ M orexin A for 30 min in the presence or absence of 10^−5^M LY294002 (Akt inhibitor) or 10^−5^ M temsirolimus (mTOR inhibitor), 10^−6^ M SB334867 (OX1R inhibitor), as well as the combination of both inhibitors. The phosphorylation of Akt (A) and mTOR (B) was normalized against the total protein expression. The total protein expression was used as an internal control for equal protein loading. Protein activation was measured by western blot. (C) Cells were treated with or without 10^−7^ M orexin A for 2 h in the presence or absence of 10^−5^M LY294002 (Akt inhibitor), 10^−5^ M temsirolimus (mTOR inhibitor), or the combination of both inhibitors. HIF-1α protein expression was measured by western blot. Data are presented as mean ± standard error of the mean based on experimental analysis in triplicate. *P< 0.05 compared to control.

It has been demonstrated that HIF-1α could be regulated at translational level via PI3K/Akt/mTOR pathway under normoxia [37, 38]. We thus evaluated the role of PI3K/Akt/mTOR in orexin A-induced HIF-1α protein accumulation in HepG2 cells. The data showed that stimulation of the cells with orexin A increased the phosphorylation of Akt and mTOR in HepG2 cells, while the total amount of Akt and mTOR were not altered (Fig 3A and 3B, respectively). Blocking OX1R with SB334867 (10^−6^ M) prior to orexin A treatment markedly reversed these effects, suggesting that orexin A induced activation of PI3K/Akt/mTOR pathway in HepG2 cells via OX1R. Moreover, administration of the LY294002 (10^−5^M, Akt inhibitor), temsirolimus (10^−5^ M, mTOR inhibitor), or a combination of both inhibitors not only suppressed Akt/mTOR (Fig 3A and 3B, respectively), but also abrogated the elevated HIF-1α protein induced by orexin A (Fig 3C). These results indicated that the PI3K/Akt/mTOR pathway participated in orexin A-mediated HIF-1α protein accumulation in HepG2 cells.

### Orexin A stimulates GLUT1 expression and glucose uptake in HepG2 cells

**Fig 4:**
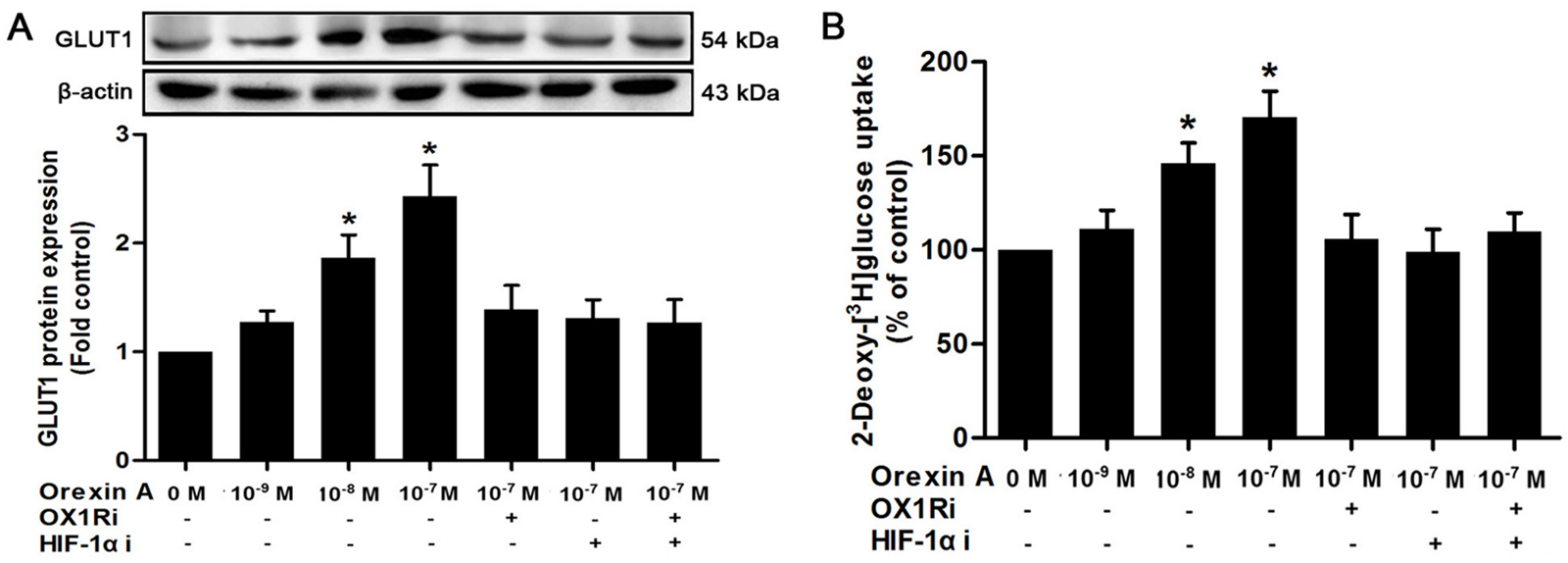
Effects of orexin A on GLUT1 protein expression and glucose uptake in HepG2 cells. Cells were treated with the indicated doses of orexin A with or without the OX1R inhibitor SB334867 (10^−6^ M), HIF-1α inhibitor, YC-1 (10^−5^ M), or a combination of both inhibitors. (A) After 2 h, GLUT1 protein expression were determined by western blot. (B) After 30 min, glucose uptake was also measured. Data are presented as mean ± standard error of the mean based on experimental analysis in triplicate. *P< 0.05 compared to control.

GLUT1 is a major contributor to glucose uptake and plays a critical role in malignant glucose metabolism. We next investigated the effects of orexin A on GLUT1 protein expression and the associated glucose uptake in HepG2 cells. Western blot showed a concentration-dependent increase in GLUT1 expression in HepG2 cells treated with orexin A. The maximal stimulation was induced by 10^−7^ M orexin A, enhancing GLUT1 expression by 2.43-fold compared with untreated cells (Fig 4A). Orexin A also stimulated glucose uptake in HepG2 cells. Stimulation with 10^−8^ M and 10^−7^ M orexin A induced a 1.46- and 1.71-fold increase in glucose uptake, respectively compared with the control (Fig 4B). To characterize the role of HIF-1α in this process, YC-1 was exploited to suppress HIF-1α pathway. Pretreatment with the HIF-1α inhibitor YC-1 (10^−5^ M), OX1R inhibitor SB334867 (10^−6^ M), or a combination of both inhibitors effectively blocked orexin A-mediated up-regulation of GLUT1 protein expression and glucose uptake in HepG2 cells (Fig 4A and 4B, respectively). These data suggested that orexin A, acting at OX1R, stimulated glucose uptake and GLUT1 expression in HepG2 cells via HIF-1α.

### Effects of orexin A on cellular ATP levels in HepG2 cells

**Fig 5:**
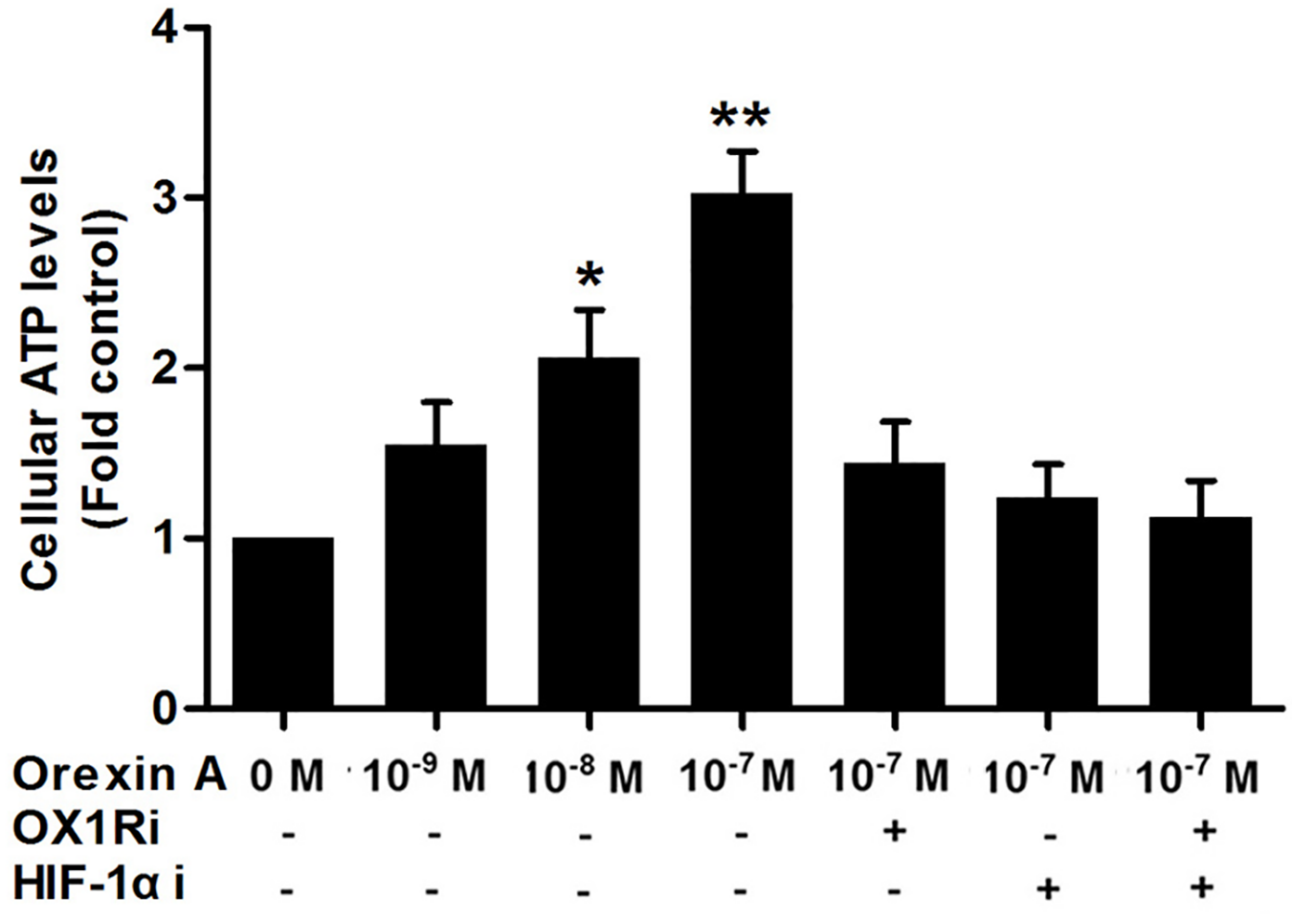
Effects of orexin A on cellular ATP content in HepG2 cells. Cells were treated with the indicated doses of orexin A for 2 h with or without the OX1R inhibitor SB334867 (10^−6^ M), HIF-1α inhibitor, YC-1 (10^−5^ M), or a combination of both inhibitors. ATP content was determined using an ATP Assay kit. Data are presented as mean ± standard error of the mean based on experimental analysis in triplicate. *P< 0.05, **P< 0.01 compared to control.

To determine whether the increased glucose uptake into cells induced by orexin A resulted in elevated energy production, we investigated the effects of orexin A on cellular ATP levels in HepG2 cells. A dose-dependent increase in ATP content was observed after 2 h of orexin A stimulation. Concentrations of 10^−8^ and 10^−7^ M orexin A led to a 2.06- and 3.03-fold increase in ATP content, respectively compared with the control. However, these effects were abrogated in the presence of the HIF-1α inhibitor YC-1 (10^−5^ M), OX1R inhibitor SB334867 (10^−6^ M), or a combination of both inhibitors (Fig 5). These findings suggested that HIF-1α participated in orexin A-induced stimulation of ATP production in HepG2 cells.

### Orexin A facilitates the metabolic flux towards TCA cycle rather than glycolysis in HepG2 cells

**Fig 6:**
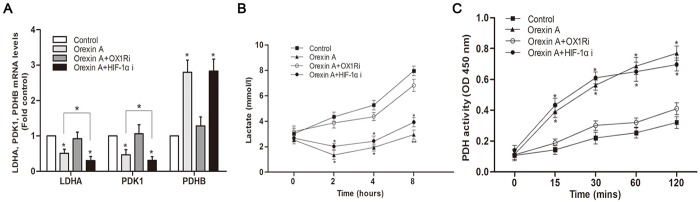
Effects of orexin A on PDHB, LDHA, PDK1 mRNA expression, lactate generation and PDH enzyme activity in HepG2 cells. Cells were treated with or without 10^−7^ M orexin A for the indicated times in the presence or absence of the OX1R inhibitor SB334867 (10^−6^ M) and HIF-1α inhibitor, YC-1 (10^−5^ M). (A) After 2 h, PDHB, LDHA, PDK1 mRNA expression were determined by real-time PCR. (B) Lactate generation was measured using a Lactic Acid assay kit. (C) PDH enzyme activity was determined using a Pyruvate dehydrogenase (PDH) Enzyme Activity Microplate Assay Kit. Data are presented as mean ± standard error of the mean based on experimental analysis in triplicate. *P< 0.05, **P< 0.01 compared to control.

Glucose is first metabolized to pyruvate through glycolysis in the cytosol. The fate of pyruvate represents a key metabolic branchpoint, as it can be converted to lactate by LDHA, thus facilitating glycolysis, or can be catalyzed to acetyl-CoA by PDH for entry into the TCA cycle in mitochondria. Pyruvate dehydrogenase kinase 1 (PDK1) phosphorylates PDH and inhibits its activity. We further investigated the intracellular fate of glucose by measuring the changes in LDHA, PDK1 and PDHB (a subunit of PDH complex) expression in HepG2 cells upon orexin A treatment. As seen in Fig 6A, orexin A stimulation resulted in a 0.50- and 0.53-fold reduction in LDHA and PDK1 mRNA levels, respectively compared with the control. In contrast, the expression of PDHB was significantly increased. Blocking OX1R with SB334867 (10^−6^ M) prior to orexin A administration significantly dampened these responses. The HIF-1α inhibitor YC-1 (10^−5^ M), however, did not abolish orexin A-induced above effects. It is noteworthy that the expression of LDHA and PDK1 were further inhibited by YC-1 in orexin A-treated HepG2 cells (Fig 6A).

We further explored whether the changes observed in the transcription data resulted in the corresponding alterations in metabolite levels. Conditioned media was collected following orexin A exposure for the indicated times and assayed for lactate production. As expected, a dramatic orexin A-induced drop in lactate production was seen at all of the time points indicated. These effects were abolished by the OX1R inhibitor SB334867 (10^−6^ M). However, pharmacological blockade of HIF-1α using YC-1 did not prevent orexin A from suppressing lactate production (Fig 6B).

The activity of PDH, a key regulatory enzyme converting glucose flux into mitochondria, was next determined in orexin A-treated HepG2 cells. We observed in accordance with our gene expression analyses that orexin A significantly increased PDH activity at all of the time points indicated. The observed increase in PDH activity was blunted when orexin A was combined with OX1R inhibitor SB334867 (10^−6^ M). However, inhibition of HIF-1α did not reverse this increase in PDH activity (Fig 6C). Together, measurements of LDHA, PDHB, PDK1 expression, lactate production and mitochondrial PDH enzyme activity indicated that orexin signaling appeared to facilitate the metabolic flux towards TCA cycle and away from glycolysis independent of HIF-1α in HepG2 cells.

## Discussion

In this study, we demonstrated the presence and cellular distribution of OX1R in HepG2 hepatocellular carcinoma cells. Orexin A promoted the expression and nuclear accumulation of HIF-1α in HepG2 cells under normoxia and the enhanced HIF-1α protein was associated with the activation of PI3K/Akt/mTOR signaling pathway. Orexin A-mediated induction of HIF-1α resulted in increased GLUT1 expression, glucose uptake as well as ATP generation in HepG2 cells. Moreover, orexin signaling directed the glucose flux towards TCA cycle rather than glycolysis. The HIF-1α pathway, although clearly responsive to orexin A, did not play an important role in mediating this metabolic branchpoint.

Orexin peptides are endogenous ligands for two receptors, OX1R and OX2R. The two receptors show broad and distinct distribution throughout the brain and peripheral tissues [7]. Many physiological actions regulated by orexins derive from highly diversified cellular responses to the orexin receptors stimulation. Recently, a growing body of evidence has demonstrated the existence of orexin receptors in cancer cells [28–35], such as human colon carcinoma cells [28], gallbladder cancer cells [33] and so on, suggesting a potential relevance of this peptide in cancer biology. Orexins and their receptors have been well-documented for their role in regulating growth and apoptosis of these cancer cells. However, the expression and metabolic function of orexin receptors in hepatoma cells remains largely unknown. In the present study, we identified the presence of OX1R in HepG2 hepatocellular carcinoma cells. Consistent with a previous study by Kishida et al [30], we did not detect the expression of OX2R in HepG2 cells. We further investigated the cellular distribution of OX1R in HepG2 cells and found that OX1R immunoreactivity was predominantly located in the nucleus. Expression of OX1R was also detected in the cytoplasm and plasma membrane. Orexin A treatment of the HepG2 cells increased the protein expression of OX1R and overall OX1R labeling. Moreover, SB334867, a specific OX1R inhibitor, effectively blocked orexin A-mediated metabolic effects in HepG2 cells, suggesting that the actions of orexin A in HepG2 cells were mediated via a direct interaction with OX1R.

Previous studies using orexin-knockout mice have demonstrated the involvement of orexin signaling in glucose metabolism [11]. Orexin deficiency in mice caused impaired glucose tolerance and insulin resistance associated with aging [11]. An *in vitro* study performed in 3T3-L1 adipocytes and isolated primary rat adipocytes demonstrated that orexin A facilitated GLUT4 translocation and stimulated glucose uptake [39]. Shen *et al*. reported that orexin A stimulated GLUT4 mRNA expression in differentiated 3T3-L1 adipocytes [40]. In addition, orexin A and its receptor acting at ventromedial hypothalamic nucleus, promoted2-deoxy-D-glucose (2DG) uptake and glycogen synthesis in skeletal muscle [41]. GLUT1 is the major contributor to the transport and metabolism of glucose in cancer cells, including HepG2 hepatic carcinoma cells [42, 43]. In the present study, orexin A significantly stimulated GLUT1 protein expression and glucose uptake in HepG2 cells. Moreover, the increased glucose utilization resulted in elevated cellular energy production as suggested by increased ATP content upon orexin A stimulation. Orexin A-mediated up-regulation of cellular energy metabolism seemed to be consistent with the involvement of orexin signaling in the promotion of energy expenditure and obesity resistance [44].

We further investigated the intracellular fate of glucose set off by orexin A. Pyruvate, as the end product of glycolysis, represents a key control point in cellular glucose metabolism. LDHA catalyzes pyruvate to lactate in the cytosol, whereas PDH processes pyruvate to acetyl-CoA for use in the TCA cycle and oxidative phosphorylation in mitochondria. PDK1 phosphorylates PDH and suppresses its activity, thus limiting influx of pyruvate into the TCA cycle. Our results showed that orexin A treatment of HepG2 cells resulted in a stimulation of PDHB expression and down-regulation of both LDHA and PDK1. It seems that orexin A signaling facilitated the glucose flux through TCA cycle and away from glycolysis. Coincide with the transcription data, orexin A stimulation resulted in a clear reduction in lactate production and significant elevation of PDH enzyme activity in HepG2 cells. These data were in line with the results of a previous study demonstrating the role of orexin in promoting the metabolic flux into mitochondrial glucose oxidation [25].

Accumulating studies show that activation of OX1R/OX2R by orexin A influences some molecules involved in intracellular metabolic function. HIF-1α, a key mediator of hypoxia response, has been revealed to play critical roles in tumor metabolism [45]. HIF-1α protein concentrations are low under normoxia as a consequence of ubiquitination and proteasome degradation by VHL [20]. During hypoxia, HIF-1α escapes from this degradation. Recent studies demonstrated that orexin A could induce HIF-1α expression via down-regulation of VHL under normoxic conditions [25, 26]. Butterick et al. suggested that orexin A-mediated regulation of energy expenditure and obesity resistance depended in part on signaling pathways involving HIF-1α [44]. In the present study, we also demonstrated that orexin A could induce HIF-1α under normoxic conditions. Our results showed that orexin A stimulated HIF-1α mRNA and protein expression as well as its nuclear accumulation in HepG2 cells. In addition to oxygen-dependent regulation, HIF-1α can also be regulated at translational level via PI3K/Akt/mTOR pathway under normoxia [37, 38]. Recent reports demonstrated that orexin A could activate the PI3K/Akt/mTOR pathway in many types of cells [39, 46, 47]. Moreover, activation of PI3K/Akt cascades was reported to induce HIF-1α protein accumulation in HepG2 cells [48]. Consistent with these findings, our data showed that orexin A induced Akt and mTOR phosphorylation in HepG2 cells, which participated in orexin A-induced up-regulation of HIF-1α. These findings indicated that orexin A-mediated induction of HIF-1α in HepG2 cells was due to both elevated HIF-1α transcript levels and an activation of PI3K/Akt/mTOR pathway, which promoted the protein expression of HIF-1α. More studies are necessary to elucidate the molecular mechanism underlying orexin A-mediated induction of HIF-1α transcription and localization.

We further investigated the relevance of HIF-1α in orexin A-mediated metabolic effects in HepG2 cells. Orexin A increased GLUT1 expression, glucose uptake as well as ATP production in HepG2 cells. However, these effects were significantly blocked by the HIF-1α inhibitor YC-1, suggesting an important role of HIF-1α in glucose metabolism. These results were in line with the general function of HIF-1α in promoting glucose uptake and glycolytic activity in cancer cells [45]. Furthermore, it is well-established that HIF-1 helps to favor the intracellular glucose flux through glycolysis, which is depended mainly on its effects in stimulating the expression of both LDHA and PDK1 [23, 49]. It can be deduced that orexin A-mediated induction of HIF-1α may result in an increase in LDHA and PDK1 expression, facilitating the pyruvate flux into glycolysis. However, measurements of both gene expression and metabolic output indicated that this was not the case in HepG2 cells treated with orexin A. Moreover, pharmacological blockade of HIF-1α did not abolish orexin A-mediated effects on LDHA, PDK1, PDHB expression, lactate content as well as PDH enzyme activity. Specially, we found an additional reduction in LDHA and PDK1 expression following YC-1 treatment in orexin A-stimulated HepG2 cells. Based on these findings, we can hypothesize that HIF-1α does have a role in stimulating LDHA and PDK1 expression, whereas in orexin A-treated HepG2 cells, there may be some other mechanisms that override HIF-1α-mediated preference for directing pyruvate flux into glycolysis. Further studied are necessary to clarify the exact molecular mechanisms behind these events. Besides HIF-1α, several regulatory pathways are also known to be involved in the regulation of glucose metabolism in tumor cells, such as Myc, PI3K/Akt/mTOR and AMP-activated protein kinase (AMPK) [17, 50]. Whether these signal molecules play a role in orexin A-mediated effects on glucose metabolism in hepatocellular carcinoma cells remains to be determined.

In conclusion, we demonstrated that orexin A oxygen-independently promoted the mRNA and protein expression of HIF-1α as well as its nuclear accumulation in HepG2 cells and the elevated HIF-1α protein was associated, at least partly, with the activation of PI3K/Akt/mTOR pathway. HIF-1α was involved in orexin A-stimulated increase in GLUT1 expression, glucose uptake as well as ATP generation. Moreover, orexin A facilitated the glucose flux into mitochondria oxidative metabolism independent of HIF-1α. Our understanding of the effects of orexin signaling on glucose metabolism in hepatocellular carcinoma remains at an early stage, further studies such as in vivo experiments are necessary to clarify this novel field. Anyway, our findings add a new dimension to the metabolic activities of this neuropeptide on hepatocellular carcinoma cells.

## Supporting information

S1 Fig The time course effects of orexin A on OX1R expression in HepG2 human hepatocellular carcinoma cells. HepG2 cells were treated with 10^−7^ M orexin A for the indicated times. OX1R protein expression was determined by western blot analysis. Data are presented as mean ± standard error of the mean based on analysis in triplicate. *P< 0.05 compared to control.
(TIF)

S2 Fig Effects of YC-1 on LDHA, PDK1 and PDHB mRNA expression in HepG2 cells. Cells were treated with or without 10^−5^ M HIF-1α inhibitor YC-1 for 2 h, LDHA, PDK1 and PDHB mRNA expression were determined by real-time PCR. Data are presented as mean ± standard error of the mean based on analysis in triplicate. *P< 0.05 compared to control.
(TIF)

S3 Fig Effects of YC-1 on lactate content and PDH enzyme activity in HepG2 cells. (A) Cells were treated with or without 10^−5^ M HIF-1α inhibitor YC-1 for 8 h, lactate generation was measured using a Lactic Acid assay kit. (B) Cells were treated with or without 10^−5^ M HIF-1α inhibitor YC-1 for 2 h, PDH enzyme activity was determined using a Pyruvate dehydrogenase (PDH) Enzyme Activity Microplate Assay Kit. Data are presented as mean ± standard error of the mean based on experimental analysis in triplicate. *P< 0.05 compared to control.
(TIF)
