# Supplementary figures and images for: Orexin A affects HepG2 human hepatocellular carcinoma cells glucose metabolism via HIF-1α-dependent and -independent mechanism
Source: PLoS One. 2017 Sep 8;12(9):e0184213. doi: 10.1371/journal.pone.0184213 (PMC5590901; doi:10.1371/journal.pone.0184213)

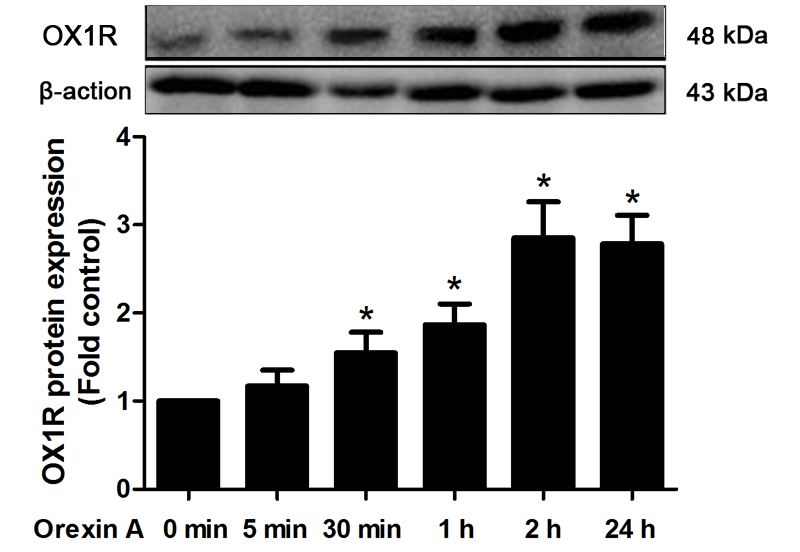

Supplement: S1 Fig — HepG2 cells were treated with 10−7 M orexin A for the indicated times. OX1R protein expression was determined by western blot analysis. Data are presented as mean ± standard error of the mean based on analysis in triplicate. *P< 0.05 compared to control. (TIF) [file pone.0184213.s001.tif]

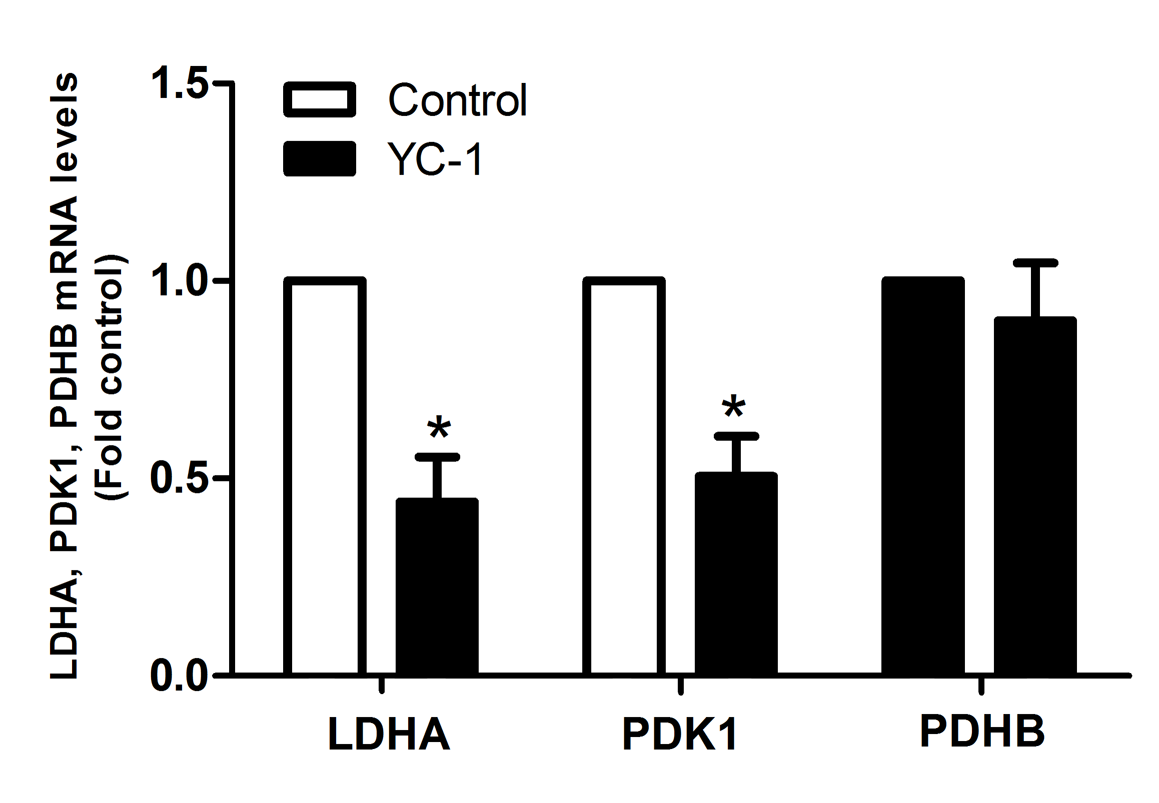

Supplement: S2 Fig — Cells were treated with or without 10−5 M HIF-1α inhibitor YC-1 for 2 h, LDHA, PDK1 and PDHB mRNA expression were determined by real-time PCR. Data are presented as mean ± standard error of the mean based on analysis in triplicate. *P< 0.05 compared to control. (TIF) [file pone.0184213.s002.tif]

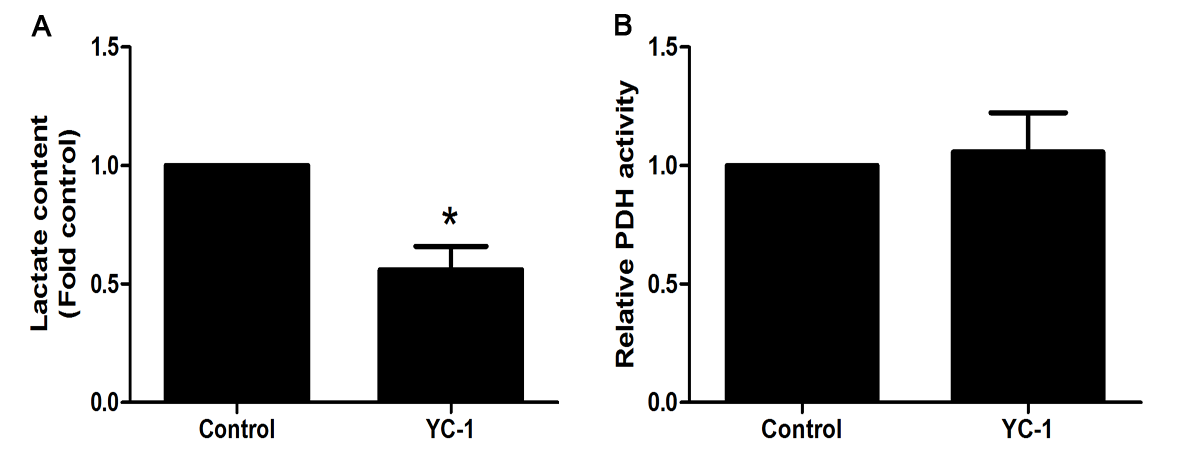

Supplement: S3 Fig — (A) Cells were treated with or without 10−5 M HIF-1α inhibitor YC-1 for 8 h, lactate generation was measured using a Lactic Acid assay kit. (B) Cells were treated with or without 10−5 M HIF-1α inhibitor YC-1 for 2 h, PDH enzyme activity was determined using a Pyruvate dehydrogenase (PDH) Enzyme Activity Microplate Assay Kit. Data are presented as mean ± standard error of the mean based on experimental analysis in triplicate. *P< 0.05 compared to control. (TIF) [file pone.0184213.s003.tif]
